# A Bioinformatics Approach to the Structure, Function, and Evolution of the Nucleoprotein of the Order Mononegavirales

**DOI:** 10.1371/journal.pone.0019275

**Published:** 2011-05-03

**Authors:** Sean B. Cleveland, John Davies, Marcella A. McClure

**Affiliations:** 1 Department of Microbiology and the Center for Computational Biology, Montana State University, Bozeman, Montana, United States of America; 2 Molecular and Cellular Toxicology Section, Laboratory of Molecular Immunology, National Heart, Lung and Blood Institute, National Institutes of Health, Bethesda, Maryland, United States of America; College of Medicine, Hallym University, Republic of Korea

## Abstract

The goal of this Bioinformatic study is to investigate sequence conservation in relation to evolutionary function/structure of the nucleoprotein of the order *Mononegavirales*. In the combined analysis of 63 representative nucleoprotein (N) sequences from four viral families (*Bornaviridae*, *Filoviridae*, *Rhabdoviridae*, and *Paramyxoviridae*) we predict the regions of protein disorder, intra-residue contact and co-evolving residues. Correlations between location and conservation of predicted regions illustrate a strong division between families while high- lighting conservation within individual families. These results suggest the conserved regions among the nucleoproteins, specifically within *Rhabdoviridae* and *Paramyxoviradae*, but also generally among all members of the order, reflect an evolutionary advantage in maintaining these sites for the viral nucleoprotein as part of the transcription/replication machinery. Results indicate conservation for disorder in the C-terminus region of the representative proteins that is important for interacting with the phosphoprotein and the large subunit polymerase during transcription and replication. Additionally, the C-terminus region of the protein preceding the disordered region, is predicted to be important for interacting with the encapsidated genome. Portions of the N-terminus are responsible for N∶N stability and interactions identified by the presence or lack of co-evolving intra-protein contact predictions. The validation of these prediction results by current structural information illustrates the benefits of the Disorder, Intra-residue contact and Compensatory mutation Correlator (DisICC) pipeline as a method for quickly characterizing proteins and providing the most likely residues and regions necessary to target for disruption in viruses that have little structural information available.

## Introduction

The Centers for Disease Control and Prevention have included the Ebola and Marburg viruses, both negative-strand RNA viruses belonging to the order *Mononegavirales*, in their list of Bioterrorism Agents/Diseases, however, structural knowledge of these agents is limited. *Mononegavirales* is composed of four viral families: *Bornaviridae* contains the *Borna Disease Virus* (BDV), which affects the nervous system and the brain in many animals, including cows and rats, and endogenous borna-like nucleoprotein elements sequences exist within the human genome [1]. *Paramyxoviridae* includes *Sendai Virus* (SENV), which typically affects rats and mice, and two viruses that cause childhood epidemics, *Measles Virus* (MeV) and *Mumps Virus* (MuV). *Filoviridae* has only two members, *Ebolavirus* and *Marburgvirus* that cause hemorrhagic fevers with mortality rates up to 90% in humans [2], [3]. The *Rhabdoviridae* contains *Rabies Virus* (RABV) and *Vesicular Stomatitis* viruses, which are both able to pass from their animal hosts to cause disease in humans, as do many *Mononegavirales*. *Vesicular Stomatitis virus* (VSV) is the model for the *Rhabdoviridae* family, and the prototype for most of the investigation of transcription and replication for the entire order of *Mononegavirales* [4]. VSV and Rabies are also used in therapies for cancer and experimental vaccines against *Human Immunodeficiency Virus* and influenza [5]–[7].

Negative-strand RNA viruses are unique in that their RNA genomes are always encapsidated by a viral coded nucleoprotein to form a ribonucleoprotein (RNP) complex. This complex serves as the template for viral RNA synthesis and forms the structural core of the viruses when packaged into virions [8]. The RNP is formed concurrently with transcription/replication by the viral RNA-dependent RNA polymerase (RdRp). For all of *Mononegavirales*, the RdRp complex is composed of the negative-sense RNA genome and three proteins: nucleoprotein (N), phosphoprotein (P) and the large subunit polymerase protein (L). The RNA genome of this complex is always found associated with the nucleoprotein as the RNP. This structure is resistant to nucleases, even during synthesis [9], [10]. The nucleoprotein, not only important for the encapsidation of the RNA for transcription, has also been identified in interactions with itself, the L polymerase and phosphoprotein for the generation of mRNAs in protein expression [11].

The nucleoprotein plays a critical role by polymerizing to cover the entire length of the genome, thereby protecting it from ribonuclease digestion [12]. This encapsidation requires association with the phosphoprotein to be chaperoned to the RNA preventing the concentration-dependent aggregation of nucleoproteins to each other. This association also keeps the N protein from encapsidating non-specific RNA transcripts during replication [13]–[15]. The nucleoproteins of bovine and human RSV viruses are able to form nucleocapsid-like structures in the absence of RNA and the other viral proteins [16], [17]. Crystal structure evidence now exists for the nucleoproteins of VSV, RABV, BDV and *Respiratory Syncytial Virus* (RSV). The VSV crystal was isolated with a 90-nucleotide strand of RNA associated with 10 copies of the nucleoprotein forming a truncated RNP in the shape of a cylinder/ring [18]. The RNA was shown to exist tightly bound in a cavity that provides a hydrophobic space to accommodate the bases of the RNA. In RSV this cavity exists within a groove at the N-N interface with seven nucleotides associated with each nucleoprotein subunit [19]. The structure of the VSV RNA-nucleoprotein complex also shows a number of interactions between neighboring nucleoproteins; each one is in contact with three neighboring N molecules forming a tetramer [20]. A comparison of the structures of the nucleoproteins of BDV, RABV and influenza A virus show that the topology of the RNA binding region from the three nucleoproteins is very similar and highlights common structural domains. The nucleoproteins each contained at least five conserved helices in the N-terminal domain and three in the C-terminal domain [21].

The current proposed mechanism for VSV RNA synthesis suggests that a portion of the nucleoprotein temporarily dissociates from the RNA allowing the polymerase access to the genome. This is supported by the crystal structure of the nucleoprotein from VSV that shows the neighboring lobe interactions provide more stability than the positively charged residues of the RNA binding cavity [22]. This work also provides evidence that structurally N would prevent access to several positions of the RNA, so no Watson-Crick base pairing could take place, and the RNP remains intact after one round of RNA synthesis, dispelling the idea that the nucleoprotein completely dissociates from the RNA during replication/transcription. Additionally, a model of RSV RNA synthesis, based on nucleocapsid-like helical assemblies, suggests that the polymerase can induce hinge movement of the N-terminal domain to the C-terminal domain. This hinge movement would result in a transient opening of the groove allowing RNA access [19].

The use of Bioinformatic methods has been implemented to produce models of the individual intra-protein contacts and disorder for the nucleoprotein in the study presented here. The results of protein disorder prediction, correlated mutations, sequence conservation, and intra-residue prediction methods have been correlated to characterize the nucleoproteins based on the data these approaches generate from the protein sequence information. The purpose of evaluating the regions of disorder within a protein is that such areas are observed to be binding sites for protein-ligand interactions. Upon association with the partner ligand the protein assumes a secondary structure as observed using x-ray crystallography [23], [24]. The flexibility that disorder imparts allows these proteins to have multiple binding partners as well as multiple functions based upon confirmation. Since the nucleoprotein interacts with the RNA genome, phosphoprotein and polymerase it is likely these regions or interaction are disordered residues that disorder prediction methods will highlight. The application of correlated mutation and intra-protein contact predictors assume that evolutionary functional constraints are expected to limit the amino acid substitution rates, resulting in a higher conservation of structural/functional sites with respect to the rest of the protein. Once a residue is changed, given the constraints operating on it, this mutation can be compensated with an additional mutation of a corresponding residue elsewhere in the protein that may be in close proximity when folded to maintain the interaction. This enables the co-evolution of the two residues that can lead to both high specificity and affinity. These assumptions can be expanded to include inter-protein residue pairs as well as protein–nucleic acid interactions [25]–[27]. The knowledge of these important residues aids in modeling protein structures when combined with additional information derived from the disorder prediction and sequence conservation. The resulting predictions provide sites that can be pursued for point mutations and inhibition within the nucleoprotein to interfere with viral transcription/replication.

## Results

### Phylogenetic Analysis

**Figure 1:**
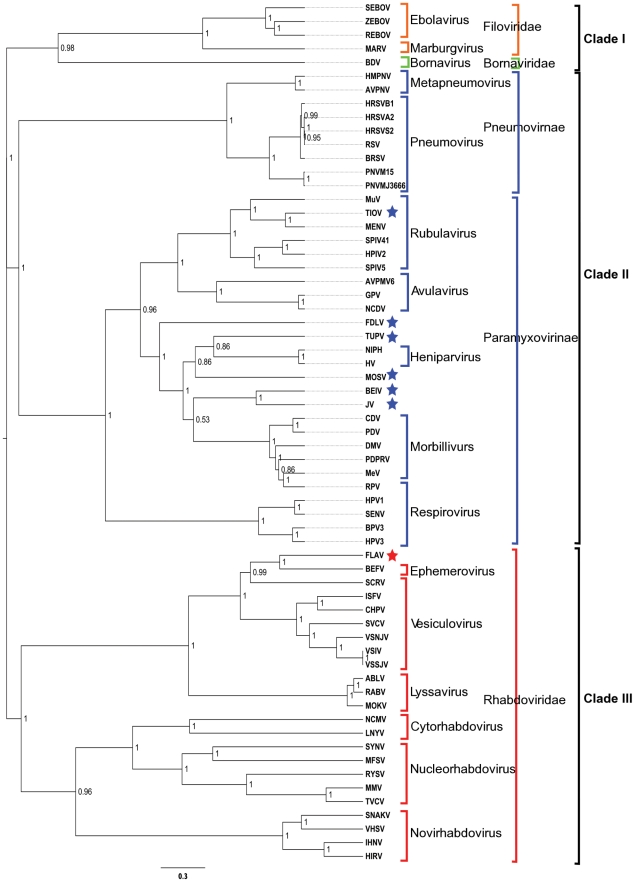
Phylogenetic reconstruction of 63 nucleoprotein sequences of the order *Mononegavirales*. The BEASTv1.5.4 tree was created using two independent Bayesian MCMC chains (10 million steps, 20% burn-in) run under the WAG amino acid substitution model [62] and rate heterogeneity among sites (gamma distribution with 4 categories). Monophyletic taxon sets consisting of *Filoviridae*, *Rhabdoviridae* and *Paramyxoviridae* were also used in the model. The posterior probabilities label each node and branch lengths are scaled to expected substitutions per site. Clade I consists of BDV and Filoviridae, Clade II contains Paramyxoviridae and Clade III is *Rhabdoviridae*. Brackets indicate virus families: *Bornaviridae*, green, *Filoviridae*, orange, *Paramyxoviridae*, blue and *Rhabdoviridae*, red. Unassigned viruses are denoted by stars colored by the family they are unassigned in.

To explore the relationship of the evolution of the nucleoprotein within the viral families and among the entire order a phylogenetic reconstruction was implemented. The multiple alignment of all 63 N sequences was generated by manual curation of a MAFFT alignment [28] that was then used as the input for MrBayes3.1 [29], [30]. The results of a MrBayes3.1 tree (results not shown) grouped BDV with the *Filoviruses*, which was different from the most recent tree created using portions of the polymerase [31]. In order to increase the confidence in this placement BEASTv1.5.4 analysis was performed and confirmed the overall MrBayes results. This tree was rooted at the midpoint and reveals three major clades (Fig. 1). Clade I is BDV and *Filoviridae*, Clade II contains *Paramyxoviridae* and Clade III is *Rhabdoviridae*; all clades show posterior probabilities (PP) of 1.

Examination of Clade I reveals that BDV clades with Filoviridae at a PP of 0.98. The *Filoviruses* group with each other and *Lake Victoria Marburgvirus* (MARV) branches from the *Ebolaviruses* at a PP of 1.

Clade II shows *Paramyxoviridae* branching into the subfamilies *Paramyxovirinae* and *Pneumovirinae* (Fig. 1). Within the subfamily *Pneumovirinae* all genera group with PPs of 0.95–1.0. *Bovine Respiratory Syncytial Virus* (BRSV) sits outside the human viruses with a PP of 1. The *Paramyxovirinae* subfamily branches into two subclades. The first contains the *Rubulavirus*, *Avulaviruses* with the unclassified *Tioman Virus* (TIOV). The *Rubulaviruses* and *Avulaviruses* relationships are highly supported by PP of 1 throughout their topology. TIOV groups within the *Rubulaviruses*. The second is made up of *Respirovirus*, *Henipaviruses*, *Morbilliviruses* and the five unclassified viruses: *Fer-de-lance Virus* (FDLV), *Tupaia Virus* (TUPV), *Mossman Virus* (MOSV), *Beilong Virus* (BEIV), and JV with a PP of 1. FDLV is an outgroup to the *Henipaviruses* and *Morbilliviruses* at a PP of 0.81. Both MOSV and TUPV group with *Henipaviruses* with PPs of 0.86 respectively. With a low PP of 0.53, BEIV and *J Virus* (JV) form their own group outside the *Morbillivirues*. The *Morbilliviruses* and *Respiroviruses* resolve relationships with PPs from 0.8–1.0.

Examination of the *Rhabdoviridae* in Clade III reveals high PPs across all genera. Within Clade III there are two subclades. The first subclade is composed of the *Ephemroviruses*, *Vesiculoviruses* and *Lyssaviruses*. The currently unassigned *Flanders Virus* (FLAV) branches with *Bovine Ephemeral Fever Virus* (BEFV) with a PP of 1 suggesting it belongs to the *Ephemeroviruses*. *Siniperca Chuatsi Rhabdovirus* (SCRV) groups between the *Ephemeroviruses* and the other *Vesiculosviruses* with a PP of 0.99. *Lyssaviruses* are an outgroup to the *Ephemeroviruses* and *Vesiculoviruses* with a PP of 1.0. The second subclade contains the *Cytorhabdoviruses*, *Nucleorhabdoviruses* and the *Novirhabdovirues*. The Novirhabdoviruses are an outgroup to the plant viruses *Cytorhabdoviruses* and *Nucleorhabdoviruses* at a PP of 0.96.

### Disorder Prediction

**Figure 2:**
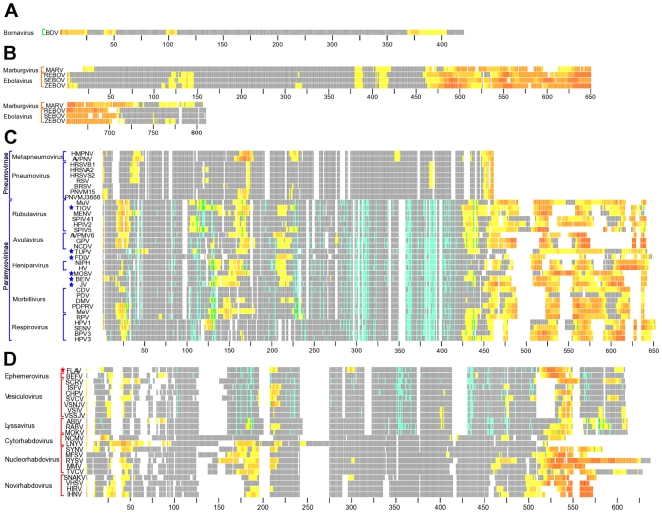
Disorder and CICP mapped residues of Family MSAs. A.) *Bornaviridae* B.) *Filoviridae* C.) *Paramyxoviridae* D.) *Rhabdoviridae*. Each family was aligned according to the process outlined in the methods section and ordered based on the results of the phylogenetic tree (Fig 1). Each residue is represented by a colored column tick corresponding to Disorder, CICP, both Disordered and CICP or neither a CICP or Disordered residue. Disordered residues are colored by an increase from yellow, being lowest confidence of disorder, to red, highest confidence of residue disorder. CICPs are shown in blue. Residues predicted to be both Disordered and a CICP are highlighted in green. Residues that have neither a Disorder or CICP prediction are represented in grey. Gaps in the alignment are represented in white. The black ticks at the bottom of the alignment denote residue position and occur every 25 residues. The color of the brackets to the left of the alignment indicate virus families: *Bornaviridae*, green, *Filoviridae*, orange, *Paramyxoviridae*, blue and *Rhabdoviridae*, red. Unassigned viruses are denoted by stars colored by the family they are unassigned in.

To identify potential residues that could be involved in inter-protein binding protein disorder prediction programs were applied to the nucleoprotein sequences and combined into a consensus prediction. The results of the four disorder predictions programs (PONDR [32]–[34], IUPred [35], [36], DisEMBL [37], and Disopred [38]) were normalized and averaged for each amino acid residue of the nucleoprotein sequences into a consensus prediction value. Those values were mapped onto the Multiple Sequence Alignments (MSAs) of each of the four viral families' nucleoproteins to observe if there is any pattern in the location of disordered regions (Fig. 2). The *Bornaviridae* sequence displays four regions of disorder with the largest being in the N and C-terminals (Fig. 2A, Table S1A). *Filoviridae* sequences contain four distinct regions of disorder with the largest being in the C-terminus. These sequences also contain the largest region of disorder of the entire order averaging over 200 consecutive residues in length beginning just downstream from residue 400 in the MSA (Fig. 2B, Table S1B).

**Figure 3:**
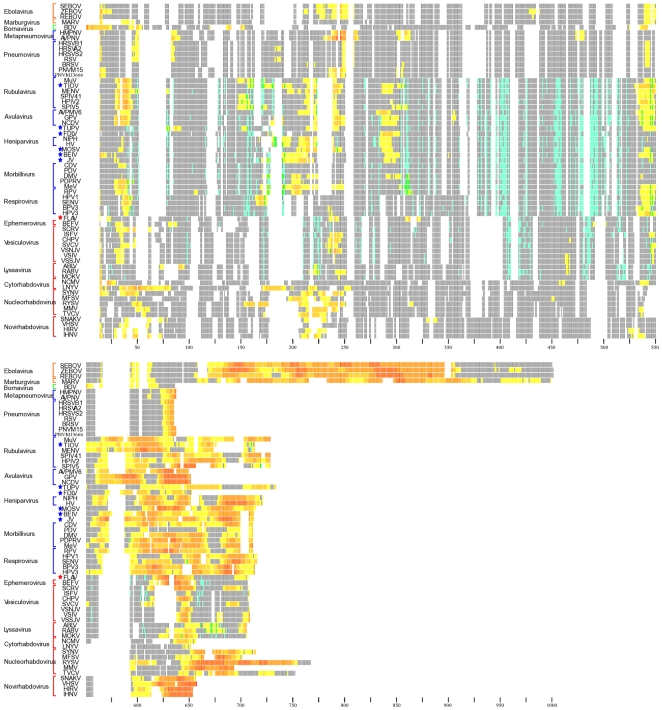
Entire Order Disorder and CICP mapped residues on the MSA. All sequences analyzed in the study were aligned using the process described in the methods and put into order according to phylogenetic tree results (Fig. 1). Each residue is represented by a colored column tick corresponding to Disorder, CICP, both Disordered and CICP or neither a CICP or Disordered residue. Disordered residues are colored by an increase from yellow, being lowest confidence of disorder, to red, highest confidence of residue disorder. CICPs are shown in blue. Residues predicted to be both Disordered and a CICP are highlighted in green. Residues that have neither a Disorder or CICP prediction are represented in grey. Gaps in the alignment are represented in white. The black ticks at the bottom of the alignment denote residue position and occur every 25 residues. The color of the brackets to the left of the alignment indicate virus families: *Bornaviridae*, green, *Filoviridae*, orange, *Paramyxoviridae*, blue and *Rhabdoviridae*, red. Unassigned viruses are denoted by stars colored by the family they are unassigned in.

*Paramyxoviridae* displays a pattern of four regions of disorder at residues ∼15–50, ∼150–180, ∼205–225, and after residue 400 in the MSA. *Paramyxovirinae* exhibits a majority of disorder beyond the 400th residue in the MSA (Fig. 2C, Table S1C). *Pneumovirinae* has a significantly smaller region of disorder in the C-terminus compared to the other sequences of *Paramyxovirinae* (Fig. 2C). *Rhabdoviridae* sequences display three regions of disorder with the largest concentration of disordered residues at the C-terminus (Fig. 2D, Table S1D). The two smaller regions of disorder are in the first half of the proteins. One is within first 100 residues of the amino terminus and the other approximately between residues 150–250 of the MSA (Fig. 2D). The *Nucleorhabdoviruses*, *Cytorhabdoviruses* and *Novirhabdoviruses* display a larger concentration of disorder in these regions compared to the rest of *Rhabdoviridae* (Fig. 2D). Disorder for the entire order's sequences exhibit three general regions of disorder with the highest concentration of consecutively disordered amino acids predicted to be at the C-terminus of the proteins (Fig. 3).

### Co-evolution and Intra-residue Contact

**Figure 4:**
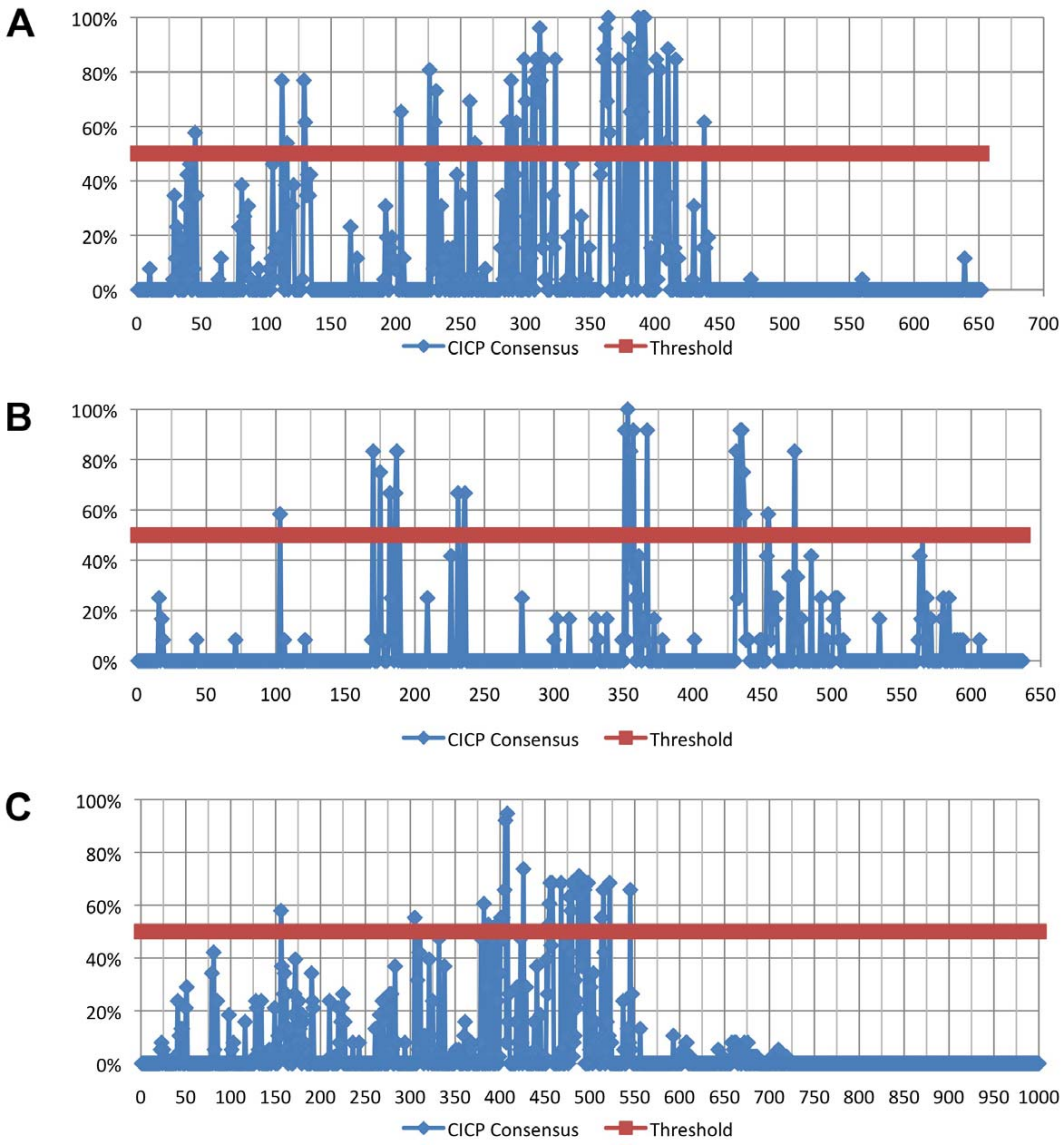
CICP Alignment Consensus Graphs. A.) Paramyxovirinae MSA. B.) Rhabdoviranae MSA. C.) Order MSA. The number of CICPs occurring for a position of the analyzed MSA was summed and divided by the total number of sequences that could participate in the CICP study from that alignment (Paramyxovirinae had 24 sequences, Rhabdoviranae has 12 sequences and the Order had 36 sequences). The y-axis is the percentage of residues predicted to be a CICP and the x-axis is the residues position in the MSA. The threshold of 50% was set to define a position as showing significant conservation of a predicted CICP and is plotted in Red. The CICP percentages are plotted in blue.

To extract information about the structural and functionally important residues that are constrained by intra-protein evolutionary pressures the results of four prediction programs were combined into a consensus prediction. The results of the two intra-residue contact predictors, ConSEQ [39], and CORNET [40], [41] were combined with the two coevolving residue mutation predictors, XDET [38], [42] and CAPS [43] and the result is referred to as the Co-evolution/Intra-residue contact prediction (CICP) consensus. CICPs were observed for 36 of the 63 viral nucleoprotein sequences from *Rhabdoviridae*, and *Paramyxoviridae* subfamily *Paramyxovirinae*, while *Bornaviridae* and *Filoviridae* could not be analyzed (Fig. 2A & B). These sequences were not analyzed due to lack of meeting the pair-wise identity criterion of 19–90%. The four prediction methods require a MSA to have a minimum of 10 sequences meeting this criterion to produce statistically significant results. The twenty-four *Paramyxovirinae* sequences that met the analysis criteria display CICPs throughout the length of the sequence. The C-terminal regions of the proteins contain few, if any, predicted CICPs in the region containing a high concentration of disordered residues (Fig. 2C). However, there is a distinct CICP pattern of highly conserved residues at positions ∼286–323 and ∼360–416, and moderately conserved residues at 225–261 throughout the Paramyxovirinae (Fig. 4A). There is a distinct area of residues that are both disordered and CICPs especially in TIOV, *Rubulaviruses*, *Henipaviruses*, BEIV, JV and *Morbilliviruses*. The residues that display disorder and CICP also correlate with hydrophobic residues and higher MSA conservation as observed in Jalview [44]. Residues ∼360–416 contain the largest number of CICPs in the sequences correlating with the highest concentration of hydrophobic residues as well as high conservation scores. Additional smaller patterns of CICPs are observed at residues ∼45 and ∼112–130 with lower percentages of conservation in the MSA. CICPs that flank a distinct region of disorder are observed at _110–130 and ∼225. Areas displaying lower frequencies of CICPs also were observed to have lower levels of hydrophobic residues and lower MSA conservation scores.

Twelve sequences meeting the analysis criteria among the *Rhabdoviridae* for *Lyssavirus*, *Ephemerovirus*, and *Vesiculovirus* could be used to estimate CICPs. The CICPs appear throughout the alignment and there is a dearth of correlation with predicted contacts in the disordered C-terminus region (Fig. 2C). There are three short regions of high CICP conservation within the MSA observed at _170–186, 351–367 and 431–473 (Fig. 4B). These contacts also correlate with pockets of hydrophobic residues and MSA sequence conservation.

Examining the MSA of the entire order reveals two regions with high concentrations of conserved CICPs at ∼382–426 and ∼447–522 (Fig. 3, 4C). These regions correlate with higher frequencies of hydrophobic residues. There does not appear to be a pattern for regions of residues predicted to be both disordered and CICPs observable outside of the *Paramyxovirinae*.

### Structural Analysis

**Figure 5:**
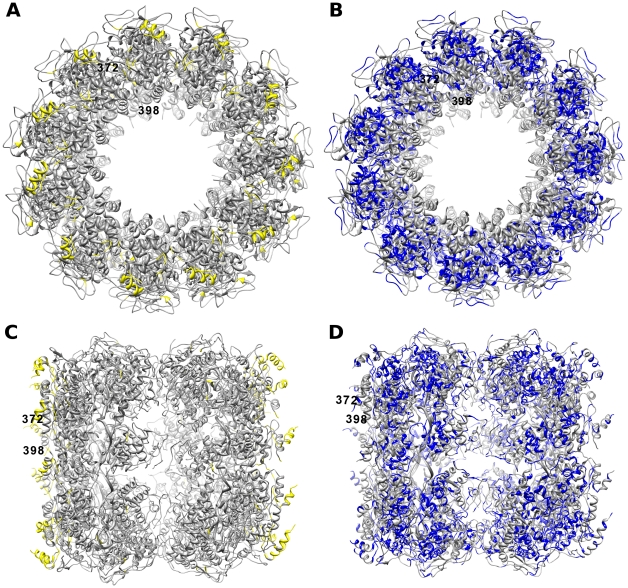
Disorder and CICP mapped Crystal structures of the *Rabies Virus* Nucleoprotein-RNA complex (2GTT). A.) Nucleoprotein-RNA ring-complex cavity view mapped with disordered residues in yellow. B.) Nucleoprotein-RNA ring-complex cavity view mapped with CICP residues in blue. C.) Nucleoprotein-RNA ring-complex side view mapped with disordered residues in yellow. D.) Nucleoprotein-RNA ring-complex side view mapped with CICP residues in blue. Structure is missing information for residues 1–6, 104–118, 185–187 and 373–397. Residues 1–2, 104–109, 378–396 are predicted to be disordered.

**Figure 6:**
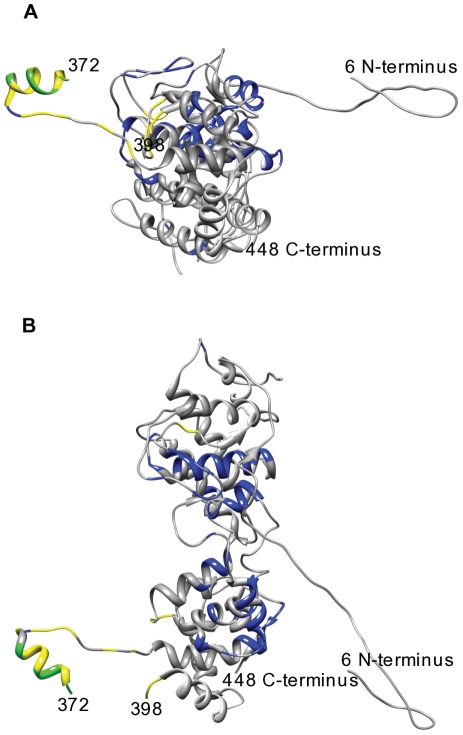
CICP and Disorder mapped Crystal structures of the *Rabies Virus* Nucleoprotein-RNA complex (2GTT) subunit-Chain A. A.) subunit-ChainA from cavity view. B.) subunit-ChainA from a side view orientation. Residues predicted to be disordered are in yellow, coevolving in blue and those predicted to be both disordered and coevolving in green. Structure is missing information for residues 1–6, 104–118, 185–187 and 373–397.

To provide a structural perspective of how the disordered regions and CICPs correlate with the nucleoprotein crystal structures solved in the last few years we mapped the results of the predictions onto these 3D structures. Using the crystal structure for the RABV nucleoprotein complex (pdb id - 2GTT) [45] from the Research Collaboratory for Structural Bioinformatics (RCSB) protein database repository with the Chimera molecular viewer [46] the disorder and CICPs were mapped to the structure by coloring the residues. Figure 5A and 5C shows the disordered regions of a RABV nucleoprotein located mainly at the periphery of the folded structure in loop regions corresponding to residues 378–401, 411–429 and 443–450 (Table S1D). Figure 5, panels B and D, highlight the CICPs that appear primarily within the interior of the protein where many residues show contact with distant residues. Figure 6 displays both the disordered and CICPs of a single nucleoprotein and shows where they overlap near the C-terminus. It should be noted that the crystal structure is missing structural information for residues 373–397, which are predicted to be disordered and residue, 383, is also predicted a CICP.

**Figure 7:**
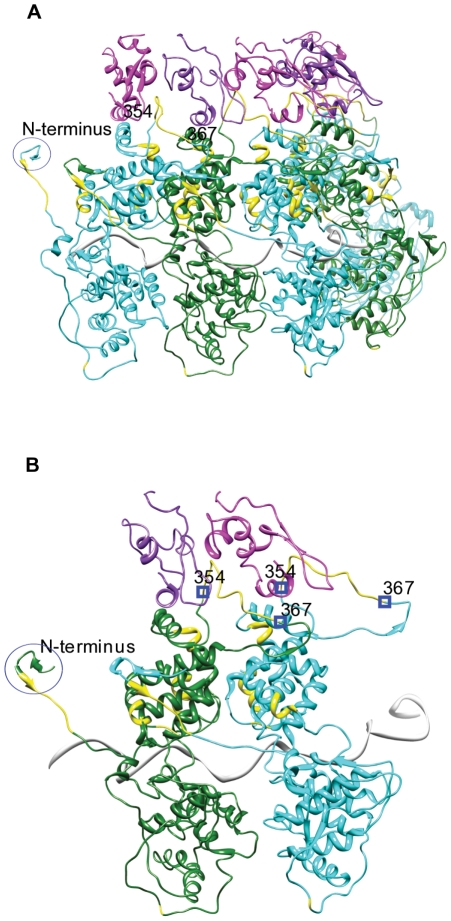
Crystal structure of *Vesicular Stomatitis Indiana Virus* nucleocapsid complexed with the phosphoprotein's nucleocapsid-binding domain(3HHW). A.) 5 nucleoproteins colored green and cyan alternating to make them easily distinguishable and 5 nucleoprotein-binding domains of the phosphoprotein colored in magenta and purple. The predicted disordered residues are highlighted in yellow. The predicted disordered nucleoprotein residues 354–367 are shown in contact with the binding domain of the phosphoprotein. B.) Two nucleoproteins and two phosphoproteins. Chain K and L are nucleoproteins colored green and cyan. Chains A and B are phosphoproteins colored magenta and purple. The blue circle is highlighting the N-terminus of the nucleoprotein and the blue squares indicate residues 354 and 367 on each N chain. Predicted disordered residues are highlighted in yellow.

For a more specific look at the nucleoprotein interaction with the phosphoprotein a recent crystal structure of the *Vesicular Stomatitis Indiana Virus* (VSIV) N∶RNA & P complex (pdb id – 3HHZ) [22] was mapped with disorder predictions for the nucleoprotein (Fig. 7). The disordered region from residues 356–369 of the nucleoprotein, chain K, appeared to be in contact with the phosphoprotein, chain A. To confirm the residues were indeed in contact a MolProbity analysis of all-atom-contact [47] was performed. The MolProbity results confirm that the phosphoprotein, chain A, residues ∼214–219 and ∼253–262 are in contact with the nucleoprotein, chain K, at residues 356–369. These correlations provide validation that the DisICC pipeline is a quick approach for suggesting which residues are involved in intra and inter-protein interactions when little is known about structure.

## Discussion

### Phylogenetic Reconstruction

The results of the BEASTv1.5.4 tree is consistent with previously published relationships of the order (Fig. 1) [31], [48]. From the tree structure it appears that BDV and *Filoviridae* are closer to each other than they are to *Rhabdoviridae* or *Paramyxoviridae* (Fig. 1). This is an interesting finding as a recent tree of the order using portions of the polymerase group BDV with *Rhabdoviridae* [31]. However, the branch length of BDV within Clade I is long indicating that it still distant from *Filoviridae*. This result, produced by both MrBayes3.1 and BEASTv1.5.4, is strong evidence that the nucleoprotein of BDV does not clade with *Rhabdoviridae*.

The *Rhabdoviridae* sequences in Clade III are organized into their respective genera as expected (Fig. 1). The relationship of FLAV with the *Ephemeroviruses* is supported by percent identity calculation of the two nucleoprotein sequences of FLAV and BEFV (36.38%), which indicate they are closer to one another than to any other sequence in the study. This result is consistent between BEASTv1.5.4 and MrBayes3.1 analyses.

The phylogenetic reconstruction of the *Paramyxovirinae* subfamily reveals some clear relationships of the previously unclassified viruses. *Menangle Virus* (MENV) and the unclassified TIOV branch together within the *Rubulavirus*. The association of MENV with the *Rubulaviruses* is supported by earlier molecular characterization and phylogenetic analysis [49]. The unclassified virus FDLV is an outgroup to the *Henipaviruses* and *Morbilliviruses*. Previous results agree with this observation as the nucleoprotein gene FDLV was shown to branch between the *Henipaviruses*, *Rubulaviruses* and *Morbilliviruses* [50]. MOSV and TUPV group between the *Henipaviruses* and *Morbilliviruses*. The relationship of MOSV and TUPV grouping is supported by previous phylogenetic work and the results from this study agree with the previous N results [51]. The nucleoprotein of BEIV and JV viruses group together between the *Henipaviruses* and *Morbilliviruses* is supported by previous phylogenetic analysis [52].

### Disorder

Disordered or intrinsically unstructured proteins (IUPs) are able to exist without a defined secondary structure. It has been shown that these IUPs can assume a secondary structure after interacting with their binding ligand. Such regions of disorder within proteins are observed to be binding sites for proteins assuming a secondary structure that is observed under x-ray crystallography when in association with the partner ligand [23], [24]. When unassociated from a binding-ligand these disordered regions are often absent from crystal structures. Disordered regions allow proteins to have many binding partners and different functions based upon the conformations. The results from the disorder predictions reveal the C-terminus of the *Mononegavirales* viral nucleoproteins contain the largest portion of disordered residues (Fig. 2E, Table S1E). This illustrates the conservation of function over sequence, as the amino acid conservation of this region is low within each of the four families and, therefore, the entire order. For example, in SENV the C-terminal amino acids, 401–524, contain the P-N binding site [9]; this region lacks residue conservation among the other *Paramyxoviruses* but does correspond with being a disordered region (Fig. 2C). NCDV was previously shown to contain a region associating with P within the first 25 amino acids of the N-terminus [53]. Similar to SENV this region lacks amino acid sequence conservation but a trend of conserved disordered residues is apparent in that region among the other *Paramyxoviruses* (Fig. 2C). Additionally, in *Newcastle Disease Virus* (NCDV) the C-terminal region at residues, 376–489, appear to be unnecessary when it comes to forming an eleven-subunit ring of the nucleocapsid, suggesting this region functions separately from the formation of the N-RNA structure [53]. Disorder prediction for NCDV shows a long disordered region encompassing that 376–389aa region highlighting a possible interaction site for the phosphoprotein (Fig. 2C). This interaction could be related to the transcription/translation process [53]. In MeV residues 477–505 have been recognized to interact with the phosphoprotein [54]. Further the disordered region of the N-tail in MeV has been shown to bind to P even when isolated from all other viral material [55]; suggesting a strong overall trend of disorder for the family of *Paramyxoviridae* in this region.

In *Rhabdoviridae* the trend is less neatly organized, as the divergence of these sequence is more than that observed in the other families, but still highlights the flexibility in the C-terminus. In addition to the C-terminal disorder observed in the other families, a region within the first 20 amino acids of the *Rhabdoviridae* sequences in the N-terminus is observed to contain disorder. In *Lettuce Necrotic Yellow Virus* (LNYV) this disordered region is larger than the corresponding disorder predictions of the other *Rhabdoviruses*, even the other *Cytorhabdoviruses* SCRV and *Sonchus Yellow Net Virus* (SYNV) (Fig. 2D). The region does correspond with the other N-terminal disordered regions of smaller size in the other viruses. Interestingly earlier in our studies the *Orchid Fleck Virus* (OFV) showed the closest match in size to this N-terminal disorder regions. OFV had been classified as a tentative *Rhabdovirus*, but has since been removed due to possessing a bipartite genome. OFV appears to go against the main trend of the other *Rhabdoviruses* and the viral order by displaying a large disordered region in the N-terminus (results not shown). As OFV is not in the family any longer these results are likely due to the existence of the OFV genome as bipartite negative-sense RNA that could require some further flexibility in function/structure compared to the non-segmented genomes. As LNYV is a single-stranded virus the similarity is either a coincidence or an undetermined link.

*Filoviridae* displays a longer region of disorder in the C-terminus compared to the other families (Fig. 2B, 3). This larger disordered region may allow the protein to maintain a similar conformation for the structural regions that are associated with RNA genome. The lack of conserved disorder within MARV compared to the three *Ebolaviruses* in region 110–140 is of note (Fig. 2B). In support of the disorder prediction from residue ∼400–670 in the *Ebolaviruses* a study observed that the amino acids 601–739 of the nucleoprotein were not required in the formation of the nucleocapsid or replication of a shortened genome; as residues 670+ are predicted to contain secondary structure it appears their function is unrelated to binding partner ligands (Fig. 2B) [3].

BDV is so different from the rest it really does not group and this is illustrated by the large disordered region in the N-terminus as compared to the majority of other viruses (Fig. 2A, 3). BDV does, however, contain a disorder C-terminal region and two additional sequence regions of disorder that are congruent with the rest of the order (Fig. 2A, 3).

### Co-evolution and Intra-residue Contact

In evolution functional constraints are expected to limit the amino acid substitution rates, resulting in a higher conservation of structural/functional sites with respect to the rest of the protein. Once a residue is changed, given the constraints operating on it, this mutation can be compensated with an additional mutation of corresponding residues across the [inter-protein] interface. This enables the co-evolution of two proteins that can lead to both high specificity and affinity. These properties can be applied to interactions such as intra-protein residue-pairs stabilizing the protein fold, inter-protein residue binding residues and protein–nucleic acid interactions [25]–[27]. The results of two intra-residue contact predictors, ConSEQ and CORNET, and two coevolving residue mutation predictors, XDET and CAPS, were combined into a consensus of structural/functional predictions. ConSEQ makes predictions by estimating the rate of amino acid evolution at each position in a MSA of homologous proteins [39]. The underlying assumption of this approach is that, in general, structurally and functionally important residues are slowly evolving. CORNET is a neural network-based method using correlated mutations, sequence conservation, predicted secondary structure, and evolutionary information [40], [41]. CAPS compares the correlated variance of the evolutionary rates at two sites corrected by the time since the divergence of the protein sequences [43]. XDET compares the mutational behavior of a residue position with the mutational behaviors of the entire alignment, which assumes the positions showing a family-dependent conservation pattern will have similar mutational behaviors as the rest of the family [38], [42]. All these methods are combined into the CICP, which correlates the structure and functional predictions with the residues that are constrained by intra-protein evolutionary pressures. The concentration of CICPs correlates with the evolutionary distances between the sequences used – the closer the evolutionary distances within a region the higher the concentration of CICPs for that region given that it also contains structural or functionally important residues.

As illustrated by the results in Figures 2C, 2D and 3 there are many residues that are predicted to be CICPs throughout the nucleoprotein sequences. Many of these residues also seem to be in contact within the protein as shown in Figures 5B, 5D and 6. These CICPs are observed to be significantly lower in frequency within the N-terminal portion of the nucleoproteins (Figs. 3, 4). This absence is most likely linked to this region being a part of the N∶N interface, which would put these residues under different evolutionary constraints of inter-protein interaction. A study of the PDPRV nucleoprotein identified that residues 1–120 and 146–241 are required for the formation and stability of the N∶N interactions [56]. These residues needed for N∶N stability correlate with the absence of highly conserved concentrations of CICPs (Figs. 2, 3, 4).

The majority of the CICPs fall in ∼382–426 and ∼447–522 within the entire order (Figs. 3, 4C), which corresponds, to residue ∼286–323 and ∼360–416 of *Paramyxoviridae* (Figs. 2C, 4A) and residue 351–367 and 431–473 of *Rhabdoviridae* (Figs. 2D, 4B). These regions are more conserved and contain more hydrophobic residues. Combined with the high concentrations of CICPs these regions appear to be important for intra-protein structural/functional interactions. While the C-terminal region has been previously shown to interact with the phosphoprotein and the first ∼240 residues of the N-terminus are part of the N∶N interface, the region ∼382–426 and ∼447–522 (Figs. 3, 4C) is well conserved containing both a high concentration of hydrophobic residues and a high frequency of CICPs. Logically such constraint would be due to the intra-protein structure and function, and possibly the interactions associated with encapsidating the RNA. This region would have less flexibility to mutate and, therefore, be conserved within the families. Contained within this region for SENV are residues 362–371, which were identified by point mutations to be essential in RNA replication [57]. The *Paramyxovirinae* show little pattern of correlation between CICPs and the concentration of disorder in the N-terminus; however, there is an overlap of residues that are correlated mutations and predicted disordered in the C-terminus residues ∼546–547 of the MSA (Figs. 3, 4C). This overlap suggests these residues may play a role in both the structure of the nucleoprotein as indicated by the CICP but also involved in inter-protein interactions at some time during the transcription/replication cycle and conformational changes that may likely involve a binding ligand interaction with the phosphoprotein or polymerase. Within the *Vesiculoviruses*, VSIV and *Spring Viremia of Carp Virus* (SVCV) (Fig. 2D, Table S1D) also display the disorder and CICP residue overlap and these residues fall into a previously identified region within RABV from residues ∼298–352 that was experimentally shown to be involved in RNA binding [58]. The RABV residues 315–319 and SENV residues 364–369 are aligned in MSA supporting functional similarity for RNA binding at this region. Further, MolProbity analysis reveals residues 287, 290, 291, 292, 312, 315 and 317 in VSIV N align within RABV residues 289–352 to be in contact with the RNA (data not shown).

### Structural Analysis

Based on the distribution of the large disordered regions of the C-terminus of the nucleoproteins being at the fringes of the nucleocapsid-ring complex (Fig. 5A, 5C) it can be inferred that these disordered regions are responsible for interacting with other nucleoproteins. When multiple units of these highlighted complexes are lined up it is obvious that a large disordered region exists that could offer access to the RNA genome encapsidated within. This disordered region could then also be defined as interfacing with the phosphoprotein, which would likely be coupled with the L polymerase to provide an interaction site for facilitating transcription or replication of the genome. This hypothesis is further supported by a previous study that found the RABV N-RNA rings had bound phosphoprotein on the tips of the rings when stained and visualized with electron microscopy [59]. More recently, a crystal structure of the VSIV N∶RNA & P complex has been solved [22] and was used to examine the mapping of the predictions to the identified binding regions in the Nucleoprotein (Fig. 7). The results of the mapping show that the predicted disordered region in the C-terminus is bound to the phosphoprotein. Further, this binding region lacks CICPs calculated for the intra-protein interactions. The presence of the disorder and absence of the intra-protein interactions in the binding region supports what we would expect biologically and, therefore, we can infer that similar characterization of the other proteins of the order *Mononegavirales* with the same disorder and CICP predictions highlights their regions of interaction.

From the evidence of this study and the corroborating findings of individual viral nucleoproteins from previous studies we can strongly infer that *Rhabdoviridae* and *Paramyxoviridae*, and more generally the other viruses in *Mononegavirales*, have similar functional/structural regions corresponding specifically to those regions showing conservation in disorder and co-evolution even though they may have weak amino acid sequence conservation. Specifically the C-terminal end of the nucleoprotein is predicated to be involved with binding to the phosphoprotein in a manner important to transcription/replication and not necessarily important to the formation of the nucleocapsid for every virus evaluated in this study. Also, it appears that evolution has constrained the function of some binding proteins not simply through sequence conservation but through conserving regions to remain disordered. These disorder and CICP residue presence and absence findings are validated by the existing experimental and crystal structure information for RABV (Figs. 5, 6) and VSIV (Fig. 7). This concordance provides confidence that the DisICC pipeline predications are valuable for sequences currently without structural information such as MuV and NIPH that both infect humans. The validation of the DisICC disorder predictions and presence or absence of CICPs with previous structural and experimental observations support our ongoing studies using predictive methods involving the other two proteins, P and L that make up the transcription/replication complex. Additionally, inter-protein prediction calculations will be performed on the each of the possible protein-protein pairs. This inter-protein contact information combined with the predicted disorder, intra-protein contacts and MSA will be used to extrapolate the definition of binding sites and residues that can be targeted for interruption to prevent viral replication.

The validation of this study by current structural information illustrates that the combination of evolutionary dynamics, disorder prediction, intra-protein structure/function predictions and co-evolving residue prediction provides the ability to identify residues and regions important for protein-ligand interactions, intra-protein interactions and protein-protein monomer interfaces. The DisICC pipeline uses sequence information to characterize proteins by predicting the residues and regions that would be necessary to target disruption in viruses that have little structural information available. As more viruses are discovered, and epidemics occur, methods such as the DisICC pipeline can quickly provide the information to aide researchers with response and development of treatments without structural information on these new and emerging viruses. For example, DisICC has the ability to produce information about protein residue positions in emerging viral strains that would point to changes resulting from new selective pressures providing researchers with possible regions to target as well as further insight into viral evolutionary strategies. The information a method like DisICC provides would also point to protein regions likely to remain unchanged as these viruses mutate thereby indicating new targets in the development of longer lived treatments. DisICC can also be applied to other multi-protein systems where identifying residues to disrupt structural/functionally conserved residues and even possible ligand binding regions without 3D structure information.

In summary, experimental and structural data validate a combined analytical approach to predicting residues and regions important for protein-ligand interactions, intra-protein interactions and protein-protein monomer interfaces. We have created the DisICC pipeline to continue our studies on the structure/function of the three proteins necessary for the replication/transcription complex of the order *Mononegavirales*. This pipeline will also add other researchers in inferring contacts among proteins complexes when little structural information is available.

## Materials and Methods

### Phylogenetic Reconstruction

The multiple sequence alignments for each family were created by submitting the sequences to the MAFFT ver.6 server (http://mafft.cbrc.jp/alignment/server/index.html) using the E-INS-i strategy. Each family alignment was manually curated to ensure optimal alignments. For the alignment of the entire order, each independent family alignment were organized into one FASTA file and submitted to the MAFFT ver. 6 alignment server using the E-INS-i strategy [26]. The MSA output was then manually curated due to the wide divergence of the sequences. This alignment was the input for MrBayes3.1 [29], [30] and BEASTv1.5.4 [60] for the generation of the phylogenetic trees. The parameters used for MrBayes3.1 were a mixed amino acid model, eight category gamma distribution rate, and 1,000,000 generations of the Markov Chain Monte Carlo analysis. In our studies, constraints were designed from our knowledge of the family classifications of the sequences resulting in four constraints. It should be noted that although the constraint parameter was invoked for the trees MrBayes3.1 overrides any constraint if the data do not support it. It has been previously explored that MrBayes3.1 with appropriate constraints, produced trees with higher confidence at each node than other tree methods: neighbor-joining, minimum evolution, maximum parsimony, and the un-weighted pair group method with arithmetic mean [61]. The outgroup used was BDV due to its difference from the other families. The BEASTv1.5.4 tree was created using two independent Bayesian MCMC chains (10 million steps, 10% burn-in) run under the WAG amino acid substitution model [62] and rate heterogeneity among sites (four category gamma distribution rate). Monophyletic taxon sets consisting of *Filoviridae*, *Rhabdoviridae* and *Paramyxoviridae* were also used in the model. The following viral proteins were included in the study: SEBOV, *Sudan Ebola Virus* (YP_138520.1); ZEBOV, *Zaire Ebola Virus* (NP_066243.1); REBOV, *Reston Ebola Virus* (NP_690580.1); MARV, *Lake Victoria Marburgvirus* (NP_042025.1); BDV, *Borna Virus* (NP_042020.1); HMPNV, *Human Metapneumovirus* (YP_012605.1); AVPNV, *Avian Pneumovirus* (AAT58236.1); HRSVB1, *Human Respiratory Syncytial Virus B1* (NP_056858.1); HRSVA2, *Human Respiratory Syncytial Virus A2* (P03418); HRSVS2, *Human Respiratory Syncytial Virus S2* (AAC57022.1); RSV, *Respiratory Syncytial Virus* (NP_044591.1); BRSV, *Bovine Respiratory Syncytial Virus* (NP_048050.1); PNVM15, *Pneumonia Virus of Mice 15* (AAW02834.1); PNVMJ3666, *Pneumonia Virus of Mice J3666* (YP_173326.1); MuV, *Mumps Virus* (NP_054707.1); TIOV, *Tioman Virus* (NP_665864.1); MENV, *Menangle Virus* (YP_415508.1); SPIV41, *Simian Parainfluenza Virus 41* (YP_138504.1); HPIV2, *Human Parainfluenza Virus 2* (NP_598401.1); SPIV5, *Simian Parainfluenza Virus 5* (YP_138511.1); AVPMV6, *Avian Paramyxovirus 6* (NP_150057.1); GPV, *Goose Paramyxovirus SF02* (NP_872273.1); NCDV, *Newcastle Disease Virus* (NP_071466.1); TUPV, *Tupaia Paramyxovirus* (NP_054690.1); FDLV, *Fer-de-lance Virus* (NP_899654.1); NIPH, *Nipah Virus* (NP_112021.1); HV, *Hendra Virus* (NP_047106.1); MOSV, *Mossman Virus* (NP_958048.1); BEIV, *Beilong Virus* (YP_512244.1); JV, *J Virus* (YP_338075.1); CDV, *Canine Distemper Virus* (NP_047201.1); PDV, *Phocine Distemper Virus* (CAA53376.1); DMV, *Dolphin Morbillivirus* (NP_945024.1); PDPRV, *Peste-des-petits-ruminants Virus* (YP_133821.1); MeV, *Measles Virus* (NP_056918.1); RPV, *Rinderpest Virus* (YP_087120.2); HPV1, *Human Parainfluenza Virus 1* (NP_604433.1); SENV, *Sendai Virus* (NP_056871.1); BPV3, *Bovine Parainfluenza Virus 3* (NP_037641.1); HPV3, *Human Parainfluenza Virus 3* (NP_067148.1); FLAV, *Flanders Virus* (AAN73283.1); BEFV, *Bovine Ephemeral Fever Virus* (NP_065398.1); SCRV, *Siniperca Chuatsi Rhabdovirus* (YP_802937.1); ISFV, Isfahan Virus (Q5K2K7); CHPV, *Chandipura Virus* (P11211); SVCV, *Spring Viremia of Carp Virus* (NP_116744.1); VSNJV, *Vesicular Stomatitis New Jersey Virus* (P04881); VSIV, *Vesicular Stomatitis Indiana Virus* (NP_041712.1); VSSJV, *Vesicular Stomatitis San Juan Virus* (P03521); ABLV, *Australian Bat Lyssavirus* (NP_478339.1); RABV, *Rabies Virus* (NP_056793.1); MOKV, *Mokola Lyssavirus* (YP_142350.1); NCMV, *Northern Cereal Mosaic Virus* (NP_057954.1); LNYV, *Lettuce Necrotic Yellows Virus* (YP_425087.1); SYNV, *Sonchus Yellow Net Virus* (NP_042281.1); MFSV, *Maize Fine Streak Virus* (YP_052843.1); RYSV, *Rice Yellow Stunt Virus* (NP_620496.1); MMV, *Maize Mosiac Virus* (YP_052850.1); TVCV, *Taro Vein Chlorosis Virus* (YP_224078.1); SNAKV, *Snakehead Virus* (NP_050580.1); VHSV, *Viral Hemorrhagic Septicemia Virus* (NP_049545.1); HIRV, *Hirame Virus* (NP_919030.1); IHNV, *Infectious Hematopoietic Necrosis Virus* (NP_042676.1).

### Disorder

Disorder calculations were performed using PONDR, IUPred [35], [36], DisoPRED2 [38] and DisEMBL [37] prediction programs. PONDR was run under the default setting and the VX-LT results were used. IUPred was run under the long sequence default settings. DisEMBL was run using default settings and the Hot-loop and Coil results were both included in our evaluation. DisoPRED2 was run under default setting. All the disorder prediction results from these methods were normalized to a 0–1 scale of disorder with values of 0.5 and greater indicating the tendency of a residue to be considered disordered. These normalized values were then combined and averaged to a consensus value using the same scale. This calculated value is used as the overall indicator for the prediction of disorder in the results. It should be noted that this consensus method provides an overall conservative prediction of disorder revealing residues with high probability of disorder and preventing over-prediction.

### Correlated Mutations and Intra-Residue Contact Prediction

The correlated mutation prediction programs used in this study were XDET [38], [42] and CAPS [43] and the intra-residue contact prediction programs implemented were ConSEQ [39] and CORNET [40], [41]. The input files for these applications were generated by calculating the pair wise percent identities within each family. MSAs of nucleoprotein amino acid sequence with less than 90% sequence identity but greater than 19% were used in the analyses. XDET, CAPS and CORNET were both run under the default parameters and ConSEQ used all defaults except the “amino acid conservation method” was set to Bayesian. The resulting predictions from each program were combined and any residues that showed a positive agreement of three or more predictors was classified as a CICP. Calculation of conservation of CICPs within the alignments is calculated per alignment position by summing up the CICP occurrences per column and dividing by the total number of sequences that participated in the CICP study for that alignment.

### Hydrophobic residues and MSA conservation

The correlation of residues in the MSAs that contained hydrophobic residues and/or high MSA sequence conservation was studied using Jalview [44]. Jalview provides visualization of hydrophobicity and sequence conversation. Conservation annotation scores were then compared with hydrophobicity for the MSA residues that displayed CICPs.

### Structural Analysis

The validity of the predictions of disorder and correlated mutations were corroborated against structural information. The existing crystal structure for the nucleoprotein complex of RABV (pdb id - 2GTT) was selected for comparison. The amino acid sequence information from the protein database file was extracted for individual nucleoprotein subunits and aligned with the corresponding amino acid sequence used in the predictions. The aligned positions were then used to map the appropriate prediction to the crystal structure with a color to highlight the corresponding residue. Chimera [46] used the prediction and alignment information to create the highlighted pdb images.

To explore predicted features that may point to protein-protein interaction the crystal structure of the VSIV N∶RNA & P complex (pdb id – 3HHZ) was used. The nucleoproteins in the complex were mapped using the same method as above. MolProbity all-atom-contact analysis [47] was conducted to verify interacting residues between the N and P proteins, and RNA interactions. The results were compared with the disordered residues and those residues reported to be in contact between N and P were reported.

## Supporting Information

Table S1 **List of predicted Disordered and Coevolving/Intra-residue contact residues for each virus.** Results are organized by family and in the same order as the phylogenetic tree (Fig. 1). The numbers in the Disorder Regions and CICP Regions columns correspond to the unaligned residue position(s) of each sequence. A.) *Bornaviridae* B.) *Filoviridae* C.) *Paramyxoviridae* D.) *Rhabdoviridae*.
(DOCX)
