# Correction: A Bioinformatics Approach to the Structure, Function, and Evolution of the Nucleoprotein of the Order Mononegavirales

**DOI:** 10.1371/annotation/6e05f8a1-c49a-4102-a8c9-188e8dc6290e

**Published:** 2011-05-26

**Authors:** Sean B. Cleveland, John Davies, Marcella A. McClure

The authors would like to add the following references:

63. Karlin D, Ferron F, Canard B, Longhi S (2003) Structural disorder and modular organization in Paramyxovirinae N and P. J Gen Virology:84 3239-3252

64. Bourhis JM, Canard B, Longhi S (2006) Structural disorder within the replicative complex of measles virus: Functional implications. Virology:344 94-110

65. Longhi, S. (2003). The C-terminal Domain of the Measles Virus Nucleoprotein Is Intrinsically Disordered and Folds upon Binding to the C-terminal Moiety of the Phosphoprotein. Journal of Biological Chemistry, 278(20), 18638-18648.

66. Jensen MR, Houben K, Lescop E, Blanchard L, Ruigrok RWH, Blackledge M (2008). Quantitative conformational analysis of partially folded proteins from residual dipolar couplings: application to the molecular recognition element of Sendai virus nucleoprotein. Journal of the American Chemical Society:130(25) 8055-8061)

67. Longhi, S. (2009) Nucleocapsid structure and function. Curr Top Microbiol Immunol 329: 103-128 

