# Correction: A Bioinformatics Approach to the Structure, Function, and Evolution of the Nucleoprotein of the Order Mononegavirales

**DOI:** 10.1371/annotation/0df2e921-af0a-45f3-975e-61c86d9574bf

**Published:** 2011-06-03

**Authors:** Sean B. Cleveland, John Davies, Marcella A. McClure

The middle initial is missing from the second author's name in the byline and citation. The correct spelling is: John S. Davies

The correct citation is: Cleveland SB, Davies JS, McClure MA (2011) A Bioinformatics Approach to the Structure, Function, and Evolution of the Nucleoprotein of the Order Mononegavirales. PLoS ONE 6(5): e19275. doi:10.1371/journal.pone.0019275 

