# Supplementary material for: A Bioinformatics Approach to the Structure, Function, and Evolution of the Nucleoprotein of the Order Mononegavirales
Source: PLoS One. 2011 May 3;6(5):e19275. doi: 10.1371/journal.pone.0019275 (PMC3086907; doi:10.1371/journal.pone.0019275)
Supplement: Table S1 — List of predicted Disordered and Coevolving/Intra-residue contact residues for each virus. Results are organized by family and in the same order as the phylogenetic tree (Fig. 1). The numbers in the Disorder Regions and CICP Regions columns correspond to the unaligned residue position(s) of each sequence. A.) Bornaviridae B.) Filoviridae C.) Paramyxoviridae D.) Rhabdoviridae. (DOCX) [file pone.0019275.s001.docx]

**Supplementary Tables:**

**Table 1A**

| **Sequence** | **Disordered Regions** | **# Disordered Residues** | **% of Sequence Disordered** | **CICP Regions** | **# CICPs** | **% of Sequence CICPs** | **Disordered and CICP** | **# Both** | **% Both** |
| --- | --- | --- | --- | --- | --- | --- | --- | --- | --- |
| BDV | 1-25, 41-51, 98-107, 319-354, | 82 | 22 |  | 0 | 0 |  | 0 |  |

**Table 1B**

| **Sequence** | **Disordered Regions** | **# Disordered Residues** | **% of Sequence Disordered** | **CICP Regions** | **# CICPs** | **% of Sequence CICPs** | **Disordered and CICP** | **# Both** | **% Both** |
| --- | --- | --- | --- | --- | --- | --- | --- | --- | --- |
| MARV | 1-13, 312-320, 333, 336-353, 393-403, 412-624, 629-630, 648-668, 670-673, 675-685, 695-695 | 304 | 43 |  | 0 | 0 |  | 0 | 0 |
| REBOV | 1-3, 120-125, 128, 131-145, 265-266, 330-339, 354, 356, 358-366, 408-473, 483-644, | 276 | 37 |  | 0 | 0 |  | 0 | 0 |
| SEBOV | 2-3, 5, 117-123, 125, 128-145, 330-338, 354, 356, 358-368, 411-644, 683, | 286 | 38 |  | 0 | 0 |  | 0 | 0 |
| ZEBOV | 1-12, 109-112, 117-125, 132-145, 262-269, 330-339, 354, 358-367, 413-474, 476-650, 683-684, 687, 697-701, 703-708, | 319 | 43 |  | 0 | 0 |  | 0 | 0 |

**Table 1C**

| **Sequence** | **Disordered Regions** | **# Disordered Residues** | **% of Sequence Disordered** | **CICP Regions** | **# CICPs** | **% of Sequence CICPs** | **Disordered and CICP** | **# Both** | **% Both** |
| --- | --- | --- | --- | --- | --- | --- | --- | --- | --- |
| HMPNV | 1, 5, 29-37, 140-155, 191-201, 297-303, 370, 379-394 | 62 | 15 |  | 0 | 0 |  | 0 | 0 |
| AVPNV | 1, 28-40, 134-156, 193-201, 297-303, 367-370, 380-394 | 72 | 18 |  | 0 | 0 |  | 0 | 0 |
| HRSVB1 | 1, 26, 28-33, 148-151, 334-338, 379-391 | 30 | 7 |  | 0 | 0 |  | 0 | 0 |
| HRSVA2 | 1-2, 25-35, 99, 101-104, 148-151, 334-338, 381-391 | 38 | 9 |  | 0 | 0 |  | 0 | 0 |
| HRSVS2 | 1-2, 26, 28-34, 148-151, 334-338, 381-391 | 30 | 7 |  | 0 | 0 |  | 0 | 0 |
| RSV | 1-2, 25-35, 99, 101-104, 148-151, 334-338, 381-391 | 38 | 9 |  | 0 | 0 |  | 0 | 0 |
| BRSV | 122, 125, 148-151, 193-194, 334-338, 380-391 | 25 | 6 |  | 0 | 0 |  | 0 | 0 |
| PNVM15 | 1-6, 138-150, 190, 192-193, 381-393 | 35 | 8 |  | 0 | 0 |  | 0 | 0 |
| PNVMJ3666 | 1-6, 138-149, 190, 192-193, 381-393 | 34 | 8 |  | 0 | 0 |  | 0 | 0 |
| MuV | 1, 16-29, 89-95, 98-106, 108, 138-152, 379-389, 405-470, 482-549 | 192 | 34 | 35, 41, 74, 78, 101, 104-107, 115-116, 148, 171-172, 205, 207, 210, 224, 230, 250, 252, 254-255, 258, 266, 271-273, 275-278, 287, 300, 304, 313-317, 325, 332-333, 335, 338-339, 341-344, 348, 351, 353, 355, 357, 363, 385, 387, | 58 | 10 | ,101,104,105,106,148,385,387 | 7 | 1 |
| TIOV | 18-29, 99-127, 142-158, 186-197, 201-207, 371-474, 477-478, 480-511, 514-517, 519-519 | 220 | 42 | 35, 41, 74, 78, 98, 101, 104-107, 115-116, 120, 171-172, 205, 207, 210, 224, 230, 234, 251, 255, 258, 266, 269, 271-273, 275-276, 278, 287, 300, 304, 313-317, 325, 332-333, 335, 338-339, 341-344, 348, 351, 355, 357, 363, 385, 387, 516, | 58 | 11 | ,101,104,105,106,107,115,116,120,205,207,385,387,516 | 13 | 2 |
| MENV | 16-32, 34-39, 123-129, 133-135, 140-156, 186-196, 372-410, 412, 418-470, 485-512, 517-519 | 185 | 35 | 35, 40-41, 59, 74, 76, 78, 98, 101, 104-107, 115-116, 148, 171, 205, 207, 210, 224, 230, 234, 255, 258, 266, 271-273, 275-278, 287, 300, 304, 313, 315-317, 325, 332-333, 335, 338-339, 342-344, 348, 351, 353, 355, 357, 363, 385, 516, | 57 | 10 | ,35,148,385 | 3 | 0 |
| SPIV41 | 1, 16-28, 90-99, 141-145, 148-149, 151-152, 373-388, 405-418, 420, 447-501, 520-543 | 143 | 26 | 35, 41, 74, 97-98, 101, 104-107, 115-116, 148, 205, 207, 210, 228, 230, 250, 254-255, 258, 266, 271-273, 275, 278, 287, 300, 313, 315-317, 325, 333, 335, 338-339, 341-344, 348, 351, 355, 357, 363, 385, | 49 | 9 | ,97,98,148,385 | 4 | 0 |
| HPIV2 | 1, 16-30, 90-100, 139-145, 147-152, 194-195, 372-388, 401-439, 445-503, 516-542 | 184 | 33 | 35, 40-41, 74, 78, 93, 98, 101, 104-107, 115-116, 204, 206, 209, 219, 229, 257, 265-266, 269-272, 274, 277, 279, 284, 286, 310, 312-317, 324, 334, 337-338, 341-343, 347, 350, 356, 362, 384, | 50 | 9 | ,93,98,384 | 3 | 0 |
| SPIV5 | 1, 16-30, 89-110, 142-152, 196-198, 200-210, 375-389, 399-434, 450-484, 486-487, 495, 497-509 | 165 | 32 | 35, 41, 74, 98, 101, 104-106, 115-116, 118, 148, 171-172, 205, 210, 224, 230, 254-255, 258, 266, 271-273, 275-276, 278, 287, 300, 313-317, 325, 333, 335, 338-339, 341-344, 348, 351, 353, 355, 357, 363, 385, 387, 504, | 53 | 10 | ,98,101,104,105,106,148,205,210,385,387,504 | 11 | 2 |
| AVPMV6 | 1, 18-28, 133-154, 181-194, 245-246, 372-389, 391, 399-465 | 136 | 29 | 38, 41, 74, 80, 101, 104-106, 115, 117-118, 148, 209, 220, 234, 255, 258, 262, 266-267, 272-273, 276, 278, 285, 287, 311-315, 317-318, 325, 330, 333, 338-339, 341-343, 348, 351, 355, 358, 363, 377, | 47 | 10 | ,148,377 | 2 | 0 |
| GPV | 1, 15-28, 115-116, 144-158, 184-191, 193-200, 242-245, 372-387, 398-444, 458-489 | 147 | 30 | 36, 38, 40, 57, 75, 77, 90-91, 98-99, 102, 174-175, 181, 183, 203, 207-208, 218, 228, 232, 249, 256, 259-260, 265-266, 268-274, 276, 283, 285, 295, 298, 309, 312-313, 315-316, 323-324, 328, 331-342, 346, 349-353, 355-356, 361, 363, 375, 383, 385, | 72 | 14 | ,375,383,385 | 3 | 0 |
| NCDV | 1, 15-27, 112-116, 143-158, 184-198, 243, 373-386, 398-446, 457-489 | 147 | 30 | 38, 40, 75, 77, 91, 98-99, 102, 175, 181, 183, 203, 207, 218, 222, 228, 232, 249, 253, 256, 259-260, 265-266, 268-269, 272-274, 276, 285, 295, 298, 309-313, 315-316, 323-324, 328, 331-342, 346, 349-351, 353, 355-356, 361, 363, 375, 383, 385, | 67 | 13 | ,375,383,385 | 3 | 0 |
| TUPV | 1, 37-48, 93-98, 139-152, 376-389, 424-500, 521-547, 551-552 | 153 | 27 | 27-28, 30-31, 36, 38, 40, 72, 91, 98-99, 102, 113-114, 116, 169, 181, 203, 207-208, 210, 218, 256, 259-260, 264-265, 268-269, 272-274, 276, 285, 302, 306, 309-313, 315-316, 323, 331-338, 340-342, 346, 355, 361, | 58 | 10 | ,38,40,98 | 3 | 0 |
| FDLV | 110, 114, 178-196, 402-431, 441-445, 447-464, 466, 471-471 | 76 | 16 | 27, 30, 36, 38, 77, 98-100, 102-103, 112, 115, 165, 176, 198, 202, 248, 255, 260, 264, 266-269, 271, 278, 280, 306-311, 318, 326-327, 330-337, 341-342, 344, 350-351, 356, 378, | 51 | 10 |  | 0 | 0 |
| NIPH | 22-23, 109, 111-124, 132-147, 182-193, 380, 395-409, 420-447, 455-473, 489-529, 532-532 | 150 | 28 | 38, 40, 72, 75, 77-78, 98-99, 102, 113-115, 151, 176-178, 181, 203, 207-208, 218, 222, 228, 232, 249, 253, 256, 259-260, 264-266, 268-270, 272-274, 276, 283, 285, 295, 298, 302, 309-313, 315-316, 323, 328, 331-342, 346, 348-350, 353, 355, 361, 375, | 73 | 13 | ,113,114,115 | 3 | 0 |
| HV | 1, 22-23, 112-123, 132-147, 182-193, 395-408, 418-475, 488-530, 532-532 | 159 | 29 | 30, 36, 38, 40, 72, 75, 77, 98-99, 102, 113-116, 146, 151, 176-178, 181, 203, 207-208, 218, 222, 228, 232, 249, 256, 259-260, 264-266, 268-270, 272-274, 276, 282-283, 285, 295, 298, 302, 309, 312-313, 315-316, 323, 328, 331-342, 346, 348-350, 353, 355, 361, 375, | 74 | 13 | ,113,114,115,116,146 | 5 | 0 |
| MOSV | 19-23, 130-133, 135-155, 184-194, 377-383, 426-471, 476-528 | 147 | 27 | 28, 30, 36, 40, 78, 98, 102, 107, 113, 116, 151, 181, 203, 205, 207-208, 218, 222, 228, 232, 249, 256, 259-260, 264-266, 269-274, 276, 285, 298, 309-313, 315-316, 323, 328-329, 331-338, 340-342, 346, 349-350, 355-356, 361, 363, 375, 383, | 66 | 12 | ,151,383 | 2 | 0 |
| BEIV | 1-7, 12, 15-16, 116-139, 187-201, 239, 372-385, 401-522 | 186 | 35 | 28, 36, 40-41, 87, 98-99, 102, 113-115, 118, 169, 181, 203, 205, 207-209, 228, 256, 259-260, 264-265, 269-274, 276, 285, 295, 298, 302, 309-313, 315-316, 323, 331-338, 340-342, 346, 352, 355-356, 361, 383, | 61 | 11 | ,118,383 | 2 | 0 |
| JV | 15-16, 18-19, 110-143, 186-192, 194-197, 382-385, 395-485, 488-522 | 179 | 34 | 35, 74, 78, 98, 101, 104-107, 113, 115-116, 170, 203, 205, 207-208, 211, 218, 232, 249, 253, 256, 260, 264-266, 269-271, 273-274, 276, 283, 285, 298, 309-311, 313-315, 323, 328, 331, 333, 336-337, 339-342, 346, 349-350, 353, 355, 361, 375, 383, | 60 | 11 | ,113,115,116,383 | 4 | 0 |
| CDV | 19-26, 395-451, 454-523 | 135 | 25 | 28, 36, 38, 40, 59, 90-91, 98, 102, 115, 117, 119-120, 171, 176-177, 183, 185, 205, 207-211, 220, 224, 230, 234, 251, 255, 258, 262, 266-267, 270-276, 278, 287, 314-318, 325, 330, 333-338, 340, 342-344, 348, 351-353, 357-358, 363, 377, 385, | 69 | 13 |  | 0 | 0 |
| PDV | 405-412, 428-435, 480-486, 507-523 | 40 | 7 | 28, 30, 36, 38, 40, 59, 72, 75, 91, 98-99, 102, 114-115, 118-120, 183, 205, 209-210, 220, 230, 255, 258, 262, 266-267, 270, 272-276, 278, 287, 312-318, 325, 330, 333-340, 342-344, 348, 351, 357, 363, 385, | 61 | 11 |  | 0 | 0 |
| DMV | 22, 113-119, 127-135, 142-149, 190-194, 197, 199-204, 209-211, 376-377, 402-413, 419-488, 502-514, 517-523 | 144 | 27 | 28, 31, 36, 38, 40, 72, 74-75, 89, 91, 98-99, 102, 115-116, 118-120, 176, 183, 205, 209-210, 220, 230, 234, 255, 258, 262, 266-267, 270-276, 278, 285, 287, 312-318, 325, 330, 333-340, 342-344, 348, 351, 357, 363, | 65 | 12 | ,115,116,118,119,209,210 | 6 | 1 |
| PDPRV | 15-31, 127-135, 138, 158, 209-211, 395-412, 418-489, 502-525 | 145 | 27 | 28, 30, 36, 40-41, 91, 98-99, 102, 107, 115-117, 119-120, 183, 205, 207, 209-211, 220, 230, 234, 255, 258, 262, 266, 270, 272-276, 278, 287, 311, 313-318, 325, 330, 333-338, 340, 342-344, 348, 351, 355, 357, 363, 418, | 61 | 11 | ,28,30,209,210,211,418 | 6 | 1 |
| MeV | 15-28, 111-160, 203-204, 207-211, 376-393, 404-408, 417-490, 503-525 | 191 | 36 | 28, 36, 38, 40, 90, 98-99, 102, 107, 115, 117, 119-120, 171, 183, 205, 207-211, 213, 220, 230, 234, 251, 255, 258, 262, 266-268, 270-276, 278, 285, 287, 314-318, 325, 330, 333, 337-338, 340-344, 348, 351-352, 355, 357-358, 363, 385, | 65 | 12 | ,28,115,117,119,120,207,208,209,210,211,385 | 11 | 2 |
| RPV | 1, 16-27, 61-64, 113-119, 127-136, 208-214, 375-389, 399-492, 508-525 | 168 | 32 | 28, 36, 38, 40, 72, 74-75, 89, 91, 98-99, 101-102, 107, 115-116, 118-120, 176, 183, 205, 207, 209-210, 213, 220, 230, 234, 255, 262, 267, 272-276, 278, 287, 312-317, 325, 330, 333-334, 336-344, 348, 351, 355, 357-358, 363, 385, | 65 | 12 | ,115,116,118,119,209,210,213,385 | 8 | 1 |
| HPV1 | 1, 21-30, 110-121, 376-389, 401-413, 436-445, 458-520, 522-524 | 126 | 24 | 28, 35, 41, 99, 102, 104, 107, 119, 123, 176, 184, 205, 210, 251, 255, 258, 261-262, 267, 270-271, 275-276, 285, 287, 311, 313-318, 325, 330, 333-340, 342-344, 348, 351, 358, 363, 385, 387, | 51 | 9 | ,28,119,385,387 | 4 | 0 |
| SENV | 1, 20-29, 111-121, 377-388, 402-414, 419-447, 460-479, 489-524 | 132 | 25 | 38, 99, 101-102, 104, 119, 122, 176, 184, 205, 210, 239, 251, 255, 258, 261-262, 267, 273-276, 285, 287, 311, 313-318, 325, 330, 333-340, 342-344, 348, 351, 355, 357-358, 363, 385, 387, 462, | 53 | 10 | ,119,385,387,462 | 4 | 0 |
| BPV3 | 18-25, 144-145, 147, 371-383, 404-515 | 136 | 26 | 6, 25, 28, 77, 86, 96, 98-101, 117, 119-120, 175-176, 183, 204, 208-209, 229, 238, 257, 260-261, 264, 266-267, 270-275, 277, 279, 284, 286, 296, 299, 310, 313-317, 324, 329, 331-343, 347, 350-351, 354, 356, 362, 376, 384, 386, | 69 | 13 | ,25,376 | 2 | 0 |
| HPV3 | 1, 19-23, 145, 147-148, 372-392, 394-446, 448-515 | 151 | 29 | 6, 25, 28, 86, 96-98, 100-101, 105-106, 116, 118-120, 170, 175, 204, 208-209, 219, 234, 260-261, 266-267, 269-275, 277, 284, 286, 310, 313-314, 316-317, 324, 329, 334-339, 341-343, 347, 354, 356, 362, 384, 386, | 58 | 11 | ,384,386 | 2 | 0 |

**Table 1D**

| **Sequence** | **Disordered Regions** | **# Disordered Residues** | **% of Sequence Disordered** | **CICP Regions** | **# CICPs** | **% of Sequence CICPs** | **Disordered and CICP** | **# Both** | **% Both** |
| --- | --- | --- | --- | --- | --- | --- | --- | --- | --- |
| FLAV | 1-5, 116-124, 167-175, 352-386, 426-436, 438-439 | 71 | 16 | 55, 90, 95, 102-103, 106-107, 109, 139, 214, 216-218, 220, 224, 230, 273-274, 276-279, 296-297, 315, 326, 333, 337, 344-345, 394, | 31 | 7 |  | 0 | 0 |
| BEFV | 10-16, 38-45, 282-284, 349-381, | 51 | 11 | 4, 73, 95, 97, 103, 106-107, 117, 134, 193, 214, 216, 219-221, 227, 237, 276-279, 295, 297, 315, 333, 386-389, 392, 395, | 31 | 7 |  | 0 | 0 |
| SCRV | 1, 16-33, 351-378, 405-421, 423, 429-429 | 66 | 15 | 11, 43, 94, 106, 110-111, 113, 121, 138, 143, 147-148, 156, 176, 178, 197, 205, 218, 220-224, 228-229, 234, 264, 277-283, 299-301, 315, 319, 330, 347, 386, | 42 | 9 |  | 0 | 0 |
| ISFV | 1, 17-20, 119-130, 317-322, 366-370, 423-423 | 29 | 6 | 57, 91, 96, 98, 103, 105-106, 111, 146, 176, 215, 217-221, 227, 231, 233, 278-279, 281-283, 285, 293-294, 300-301, 336, 346-347, 349, 377, 379, 381-382, 418, | 38 | 8 |  | 0 | 0 |
| CHPV | 1, 19-20, 28-29, 117-128, 266-267, 352-372, 422-422 | 41 | 9 | 57, 92, 97, 104, 108-109, 136, 141, 145-146, 197-198, 217, 219-224, 228, 233, 276, 279-281, 314, 318, 320, 345, 348, 352, 377-378, | 33 | 7 | ,352 | 1 | 0 |
| SVCV | 1, 16-27, 112, 114-122, 315-323, 343-351, 353, 359-365, 397-409, | 62 | 14 | 54, 57, 89, 94, 101, 105-106, 108, 133, 138, 142-143, 151, 214, 216, 220, 224-225, 228-230, 273, 276-278, 281, 311, 315, 373, 375, 378, | 31 | 7 | ,315 | 1 | 0 |
| VSNJV | 1-2, 13-21, 116-128, 317-320, 360-371, 422-422 | 41 | 9 | 56, 91, 96, 103, 107-108, 118, 135, 140, 144-145, 153, 216, 218, 220, 222, 232, 275, 278-281, 298, 317, 328, | 25 | 5 | ,118,317 | 2 | 0 |
| VSIV | 1, 15-21, 121, 261-263, 265, 319-320, 356-369, 392-396, 416-422 | 41 | 9 | 56, 91, 96, 103, 108, 140, 144-145, 184, 216, 218, 220, 222, 232, 275, 278-281, 298, 317, 328, | 22 | 5 |  | 0 | 0 |
| VSSJV | 1, 15-21, 121, 261-263, 265, 319-320, 356-369, 392-396, 416-422 | 41 | 9 | 56, 91, 96, 103, 108, 140, 144-145, 184, 216, 218, 220, 222, 232, 275, 278-281, 298, 317, 328, | 22 | 5 |  | 0 | 0 |
| ABLV | 2, 37-46, 104-108, 273-274, 276, 391-406, 409-423, 443, 445-450 | 57 | 12 | 8, 10, 92, 97, 104, 108-109, 111, 227, 229-231, 233, 243, 286, 289-290, 308-310, 312-315, 324, 328-330, 332, 356, 388, 406, 416-417, 421, 425, | 36 | 8 | ,104,108,406,416,417,421 | 6 | 1 |
| RABV | 1-2, 103-109, 124-134, 273-274, 276, 378-401, 411-429, 443-450 | 74 | 16 | 11, 92, 108, 111, 215, 229-234, 236-237, 240, 243, 248, 286-288, 290, 308, 313, 315, 330, 332, 355, 357, 411, 416-417, 421, 427, 431, | 33 | 7 | ,108,411,416,417,421,427 | 6 | 1 |
| MOKV | 1-2, 127-128, 371-403, 450-450 | 38 | 8 | 8, 22, 92, 97, 109, 146, 151, 227, 229, 231, 233, 243, 248, 286, 289-290, 308-310, 312-315, 328, 330, 358, 388, 406, 417, 421, 429, | 31 | 6 | ,388 | 1 | 0 |
| NCMV | 1-11, 315-316, 318-320, 367-375, 410-428, 430-431 | 46 | 10 |  | 0 | 0 |  | 0 | 0 |
| LNYV | 1-3, 6-7, 19-66, 121-143, 148, 150, 165-173, 193-199, 452, 454-456, 458-459 | 100 | 21 |  | 0 | 0 |  | 0 | 0 |
| SYNV | 1-8, 17-34, 122-133, 139-155, 419-463, 465-471, 473-475 | 110 | 23 |  | 0 | 0 |  | 0 | 0 |
| MFSV | 33, 117-137, 142-151, 314-316, 373, 375, 409-422, 424-460, 462-462 | 89 | 19 |  | 0 | 0 |  | 0 | 0 |
| RYSV | 26-35, 101-150, 199-202, 354-377, 396-508, | 201 | 38 |  | 0 | 0 |  | 0 | 0 |
| MMV | 1, 25-37, 123-146, 345-355, 397-447 | 100 | 22 |  | 0 | 0 |  | 0 | 0 |
| TVCV | 1, 30-33, 114-139, 347-348, 350, 394-403, 409, 411-416, 421-447, 467-482, 495, | 95 | 18 |  | 0 | 0 |  | 0 | 0 |
| SNAKV | 19-30, 34, 99-108, 160-166, 341-349, 351-352, 360-399 | 81 | 20 |  | 0 | 0 |  | 0 | 0 |
| VHSV | 1-2, 18-27, 345-404 | 72 | 17 |  | 0 | 0 |  | 0 | 0 |
| HIRV | 1-2, 12-24, 102-115, 311-318, 344-392 | 86 | 21 |  | 0 | 0 |  | 0 | 0 |
| IHNV | 1-3, 13-31, 98-112, 315, 317-324, 342-391 | 96 | 24 |  | 0 | 0 |  | 0 | 0 |
